# Analysis of distributions reveals real differences on dichotic listening scores between left- and right-handers

**DOI:** 10.1093/texcom/tgad009

**Published:** 2023-06-01

**Authors:** Emma M Karlsson, Kenneth Hugdahl, Marco Hirnstein, David P Carey

**Affiliations:** Institute of Cognitive Neuroscience, School of Human and Behavioural Sciences, Bangor University, Bangor, United Kingdom; Department of Experimental Psychology, Ghent University, Ghent, Belgium; Department of Biological and Medical Psychology, University of Bergen, Bergen, Norway; Department of Biological and Medical Psychology, University of Bergen, Bergen, Norway; Institute of Cognitive Neuroscience, School of Human and Behavioural Sciences, Bangor University, Bangor, United Kingdom

**Keywords:** cerebral lateralization, dichotic listening, handedness, hemispheric asymmetry, language

## Abstract

About 95% of right-handers and 70% of left-handers have a left-hemispheric specialization for language. Dichotic listening is often used as an indirect measure of this language asymmetry. However, while it reliably produces a right-ear advantage (REA), corresponding to the left-hemispheric specialization of language, it paradoxically often fails to obtain statistical evidence of mean differences between left- and right-handers. We hypothesized that non-normality of the underlying distributions might be in part responsible for the similarities in means. Here, we compare the mean ear advantage scores, and also contrast the distributions at multiple quantiles, in two large independent samples (Ns = 1,358 and 1,042) of right-handers and left-handers. Right-handers had an increased mean REA, and a larger proportion had an REA than in the left-handers. We also found that more left-handers are represented in the left-eared end of the distribution. These data suggest that subtle shifts in the distributions of DL scores for right- and left-handers may be at least partially responsible for the unreliability of significantly reduced mean REA in left-handers.

The link between the left hemisphere and speech was discovered by the behavioral neurology of Broca, Dax, and Wernicke long before more current technologies, like functional magnetic resonance imaging (fMRI; e.g., Hugdahl et al. 1999; Friston 2004) enabled non-invasive measurement of asymmetric brain processes. In between these two bookends of hemispheric specialization research came a bronze age of inexpensive, non-invasive methods of documenting cerebral asymmetry. This era gave rise to many technologically innovative experiments utilizing techniques such as visual half field presentations (Bryden 1965; Hugdahl and Franzon 1985), and of course, dichotic listening (Kimura 1961a).

In dichotic listening paradigms, two different stimuli are presented simultaneously, one to each of the ears. This paradigm was originally developed by Broadbent (1952) for studying attention switching, but later used by Kimura (1961a, 1961b) to examine hemispheric specialization in the perception of speech. The assumption is that the right ear advantage (REA) found in the majority of individuals is an indication of left-hemispheric specialization for speech perception. More specifically, it is assumed that verbal stimuli presented to the left ear are chiefly projected to the right hemisphere (due to more prominent contralateral auditory pathways) before being transferred back to the left hemisphere. This extra transfer would lead to a signal or time loss giving rise to the REA (Kimura 1967).

Refinements from early experiments that used crudely recorded words or sentences, lead to improvements culminating in digitally produced and quantified consonant-vowel (CV) pairs, in which most participants blend sufficiently well, minimizing stimulus dominance or ear signature problems (Westerhausen 2019). Sadly, in parallel with improvements in design and analysis, questions related to brain asymmetry and handedness are becoming less frequent, replaced by questions related to cognitive control, in some sense moving towards the original intentions of Broadbent (Hugdahl et al. 2009; Westerhausen and Kompus 2018). Another reason that the focus on hemispheric specialization has faded from the dichotic listening literature to some extent is the emergence of newer technologies such as fMRI. However, dichotic listening only requires headphones and a digital device, is quick to administer, and is well suited for large sample efforts with university undergraduates, younger people, and individuals for whom scanning is unpleasant or impossible (Stipdonk et al. 2022). Online versions can provide very good test–retest reliabilities as well (Parker et al. 2020).

CV dichotic listening paradigms tend to produce reliable ear advantages in individuals (Speaks et al. 1982; Hugdahl 2011; Parker et al. 2020; Westerhausen and Samuelsen 2020). Whilst the obtained ear advantages are often small, they have some face validity. Dichotic listening scores have been validated against more direct measures of asymmetry using Wada testing and fMRI (Hugdahl et al. 1997; Hund-Georgiadis et al. 2002; Westerhausen et al. 2014), and produce large ear differences in patients with cerebral commissurotomies (Sparks and Geschwind 1968; Pollmann et al. 2002). These findings suggest that dichotic listening measures cerebral asymmetry, for some aspects of language, at least.

One interesting caveat is that group mean differences in dichotic listening ear advantages between right-handed and left-handed participants are often smaller than expected, considering the reduced number of left-handed individuals with left-hemisphere specialization for speech and language (Carey and Johnstone 2014). Approximately, 70% of left-handers are left hemisphere dominant for speech, as compared to approximately 95% of right-handers according to Wada tests (Rasmussen and Milner 1977). This large bias in both groups is rarely commented on by handedness researchers. In fact, more often than not, right-handers and left-handers do not differ statistically on dichotic listening tests, although the numerical differences are inevitably in the predicted direction of reduced REA in the left-handed participants (Briggs and Nebes 1976; Hugdahl et al. 2009; Hirnstein et al. 2014). When this difference achieves statistical significance, the effects tend to be small and depend on large samples (Bless et al. 2015; in Karlsson et al. 2019 compare E1 and E2).

If verbal dichotic listening ear biases are largely the result of hemispheric specialization for verbal stimuli, then REAs should be found more frequently in the right-handers compared to left-handers. In fact, if the estimates of hemispheric specialization for language, as a function of handedness, from Wada testing are accurate, differences in the left-ear end of the distributions should be even more pronounced, favoring left-handed membership; left-handers are three times more likely to have right hemisphere specialization (15 versus 5%); while right-handers are only 1.4 times more likely to have left language specialization ((95 versus 70%); Carey and Johnstone 2014). As dichotic listening is an indirect measure of hemispheric specialization, it must misclassify some individuals, but these effects should be similar in left-handed and right-handed groups (e.g. differences in sensitivity to speech-related frequencies in each ear; inadvertent attempts to listen to one ear or the other, and so on). Therefore, given known percentages of hemispheric specialization for language as a function of handedness, there should be more left-handers with an LEA compared to right-handers, and more right-handers with an REA as compared to left-handers (Carey and Johnstone 2014).

However, a recent re-analysis of three previously published dichotic listening experiments, Packheiser et al. (2020) claim that there is virtually no statistical relationship between handedness as assessed by the Edinburgh handedness inventory (EHI) and dichotic listening laterality. They examined this relationship by correlating dichotic listening scores with EHI scores in their 1,554 participants (137 left-handers if an EHI score of < 0 is used to classify individuals (Personal communication, J Packheiser, 2020 March 29.)). Although they found a weak, but significant, correlation between the two measures, a Bayesian correlation matrix analysis indicated “anecdotal evidence” for the lack of a relationship between the two measures.

One as yet unexamined possibility for the inconsistent effects of handedness (for mean differences in particular) is that the distributions of ear advantage scores are not completely normal in these two groups. The historical reliance in neuropsychology on parametric significance hypothesis testing, reduced sensitivity in participants with an intact corpus callosum, and non-normal distributions may go a considerable distance in explaining these paradoxically small effects of handedness.

The purpose of the present study is to compare the distributions of dichotic listening laterality indices (LIs) in large samples of right-handers and left-handers, using two large independently-collected datasets, focusing on characteristics of the distribution of the scores, and not just their means. In the first sample, collected in Bergen, sampling was random with respect to handedness, so the frequency of left-handers relative to right-handers is lower than in the second sample, collected in Bangor. In this latter study, left-handers were actively recruited (see Karlsson et al. 2019). We elected to characterize the sampling distribution of the CV dichotic listening scores in considerable detail by utilizing the shift function approach recommended by Rousselet et al. (2017). This latter approach provides an easy to understand and statistically rigorous procedure to examine different handedness group membership across the entire distribution of scores. Our working hypothesis was that handedness group membership might only differ in the tails of the distribution and therefore not always be detectable by measures of central tendency. Specifically, it was hypothesized that more left-handers would be represented in the left-ear advantage end of the distribution.

## Materials and methods

### Participants

#### Bergen sample

Data from 1,358 participants were collected from the Bergen group and collaborators as part of a long-standing series of studies on top-down and bottom-up processes. The Bergen database is the largest database of dichotic listening scores to date. This sample included data from 1,232 right-handed (666 female, 566 male) and 126 left-handed (80 female, 46 male) participants aged 16 or older. Exact age is not known for 407 (30%) of the participants, as they were initially allocated to age groups. In the sample, 1,144 participants were aged between 16 and 49, and 214 participants were 50 or older. Handedness was assessed with either the Edinburgh Handedness Inventory (Oldfield 1971) or the Raczkowski questionnaire (Raczkowski et al. 1974). Participants were classified as right-handed if they carried out most activities from the inventories with their right hand, or left-handed if they showed no hand preference or had a preference for their left hand. Hearing thresholds were determined for most participants, and all of these could detect frequencies of up to 3,000 Hz at an intensity of 20 dB in both ears, and had an interaural acuity difference of no more than 10 dB. Exclusion criteria for the study included participants with hearing deficits or a history of psychiatric or neurologic diagnosis.

#### Bangor sample

Participants were 1,042 Bangor University students, staff members, and members of the general public recruited opportunistically and via a student participation panel. Out of the participants, 588 were right-handed (392 females, 195 males, gender data was missing from one participant), as assessed with a modified Waterloo Handedness Questionnaire (WHQ; Steenhuis and Bryden 1989). The 454 left-handers (306 females, 148 males) were left-handed as assessed with the WHQ or reported being forced to switch to writing with their right hand. These data were taken from a dataset that is continuously updated by the Bangor group as part of an ongoing project on hemispheric asymmetries, and includes 411 additional participants to those reported in Karlsson et al. (2019).

### Stimuli

#### Bergen

The consonant–vowel syllables are paired presentations of the six stop-consonants /b, d, g, p, t, k/ with the vowel/a/to form six consonant–vowel (CV) syllables: /ba/, /da/, /ga/, /pa/, /ta/, /ka/ (Hugdahl et al. 2009). These were combined in pairs and played in each sound channel (e.g., /pa/−/ga/), resulting in 36 stimulus pairings including six homonyms (e.g., /ba/—/ba/). The stimuli were presented three times in three separate blocks (108 trials in total). Each block contained all possible syllable pairings including homonyms. The three-block version of this task is traditionally used to measure cognitive control by directed attention, comprising of three conditions; a “non-forced attention” condition and two “forced right/ left” conditions, where participants are specifically asked to report the stimulus from the right or left ear (Hugdahl and Andersson 1986; Hugdahl et al. 2009). Only the first block of 36 trials given under the non-forced condition was included for analysis. The six trials of homonyms were excluded from laterality calculations.

#### Bangor

The stimuli for the CV dichotic listening (DL) paradigm were the same as for Bergen. However, in this sample, all three blocks were given under non-forced conditions to calculate an ear advantage score from all 90 trials (excluding the 18 homonyms), driven by an interest in predicting hemispheric specialization on an individual basis.

### Procedure

#### Bergen

Stimuli were either presented via analog or digital tape/CD players or on a PC (using E-Prime software; Psychology Software Tools Inc., Pittsburgh, PA), with a sound intensity of about 70 dB SPL, depending on the laboratory. The instructions to participants were to report back the one syllable they heard best and most clearly, they were not informed that there were two different syllables presented in each trial. The response was either given orally by the participant, and recorded by the experimenter, or entered by the participant using a button press.

#### Bangor

Participants were given a set of headphones and were instructed they would hear a pair of syllables presented in each trial. They were instructed to report back the syllable they heard or if they heard two different sounds, the one they heard best or most clearly. They were instructed to center their attention to their best ability and not focus their attention by listening to the syllables presented to a particular ear. The participants were also told that they may not report all syllables an equal number of times, and not to worry if they reported the same syllable several times in a row. The participants were encouraged not to spend time thinking about the sounds, but to report one back as soon as the sound had been presented by verbally reporting the sound and to point to it on a response sheet that was given at the start of the experiment. The experimenter entered the response using the keyboard, which triggered the next trial. A rest period was offered between each block.

### Analysis

**Fig. 1:**
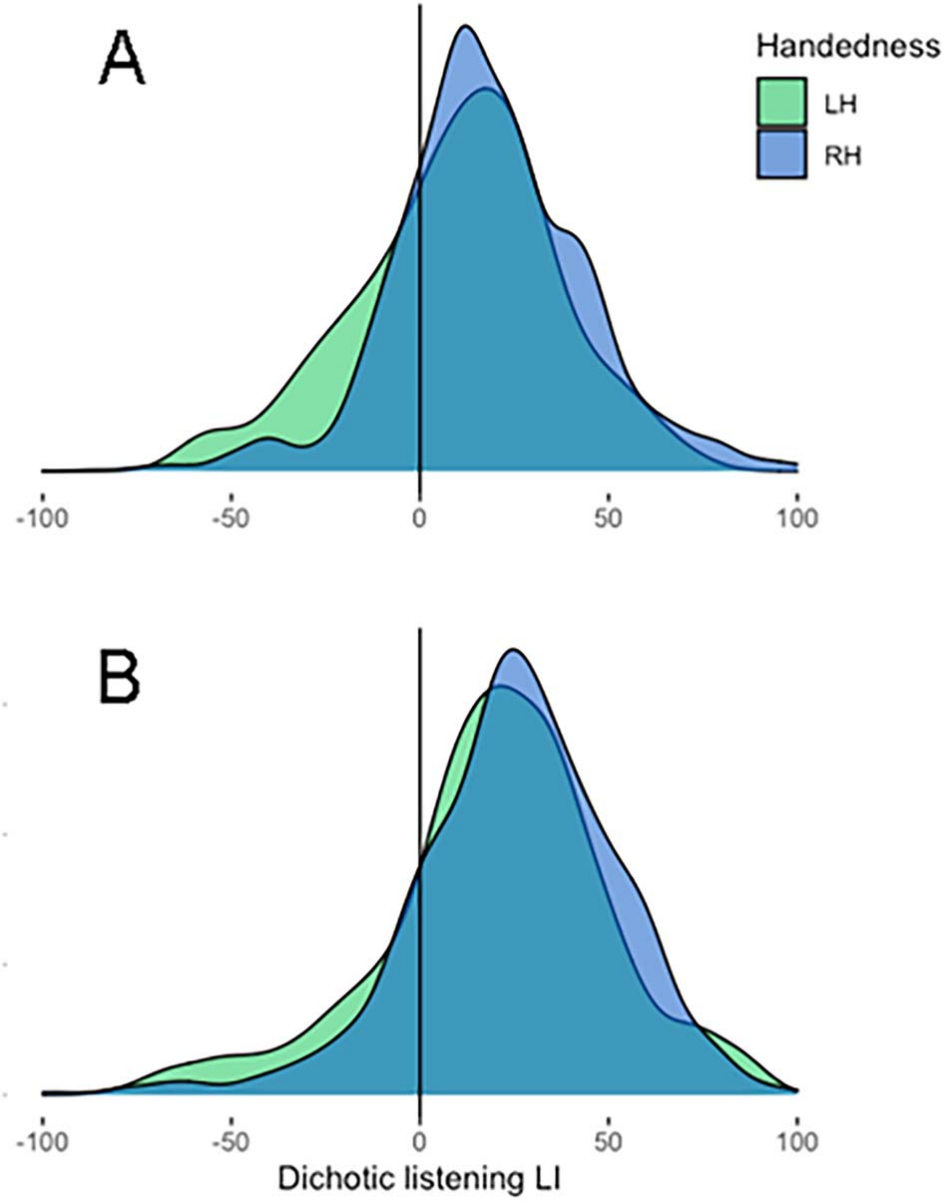
Overlapping density curves of left-handers (LH; green) and right-handers (RH; blue) dichotic listening LI scores for (A) Bergen and (B) Bangor. The overlap between the two handedness group distributions can be seen as turquoise in both graphs.

A laterality index (LI) was calculated for each participant, LI = (R − L)/(R + L) × 100, where R/L equals the number of correctly identified stimuli from the right/left ear. Response bias scores ranged from −100 to +100, with negative scores reflecting a left-ear advantage (LEA) and positive scores reflecting a right-ear advantage (REA). The histograms for all LIs as a function of handedness group in the two separate datasets appear in Fig. 1. The first set of analyses concentrated on the typical comparison of group means for each handedness group in the Bergen and Bangor samples. Secondly, the approach by Rousselet et al. (2017) was utilized, which is based on a shift function**.** Shift functions afford comparisons of data from two groups and to assess if these differ at several points in the two distributions (Wilcox 1995; Rousselet et al. 2017). In the present study, the shift function was used to quantify differences between all deciles to illustrate how much one distribution needs to be shifted to match that of another. The shift function was implemented in the “Rogme” R package (Rousselet and Wilcox 2016) which uses the Harrell–Davis quantile estimator to estimate the deciles of two distributions (Harrell and Davis 1982), and computes 95% confidence intervals of the decile differences. The 95% bootstrap confidence intervals were computed from 2,000 samples.

## Results

### Bergen dichotic listening LI scores

Probability density functions of dichotic listening LIs for both handedness groups in both samples appear in Fig. 1. Using one-sample *t*-tests against 0, an REA was found for both right-handers (*M* = +18.04, *SD* = 25.15, 95% CI +16.63, +19.45), *t*(1,231) = 25.18, *P* < 0.001, and left-handers (*M* = +9.68, *SD* = 26.35, 95% CI +5.03, +14.33), *t*(125) = 4.13, *P* < 0.001. The REA was significantly greater in right-handers as compared to left-handers, *t*(1,356) = 3.54, *P* < 0.001, *d* = 0.32, 95% CI of the mean difference (8.36) did not overlap with zero [3.51, 13.21]. As we have recommended elsewhere (Carey and Johnstone 2014; Karlsson et al. 2019), the percentages showing a right-ear advantage (REA) in each group were also compared. Seventy-one right-handers and four left-handers had no ear advantage and were excluded from this analysis. Eighty-one percent of the right handers (940/1,161) and 69.7% of the left-handers (85/122) had rightward biases, and this difference of 11.3% was significant as compared with a one-tailed z test, *z* = 2.96, *P* = 0.002, *h* = 0.26, 95% CI of the difference [3.5%, 20.2%].

**Fig. 2:**
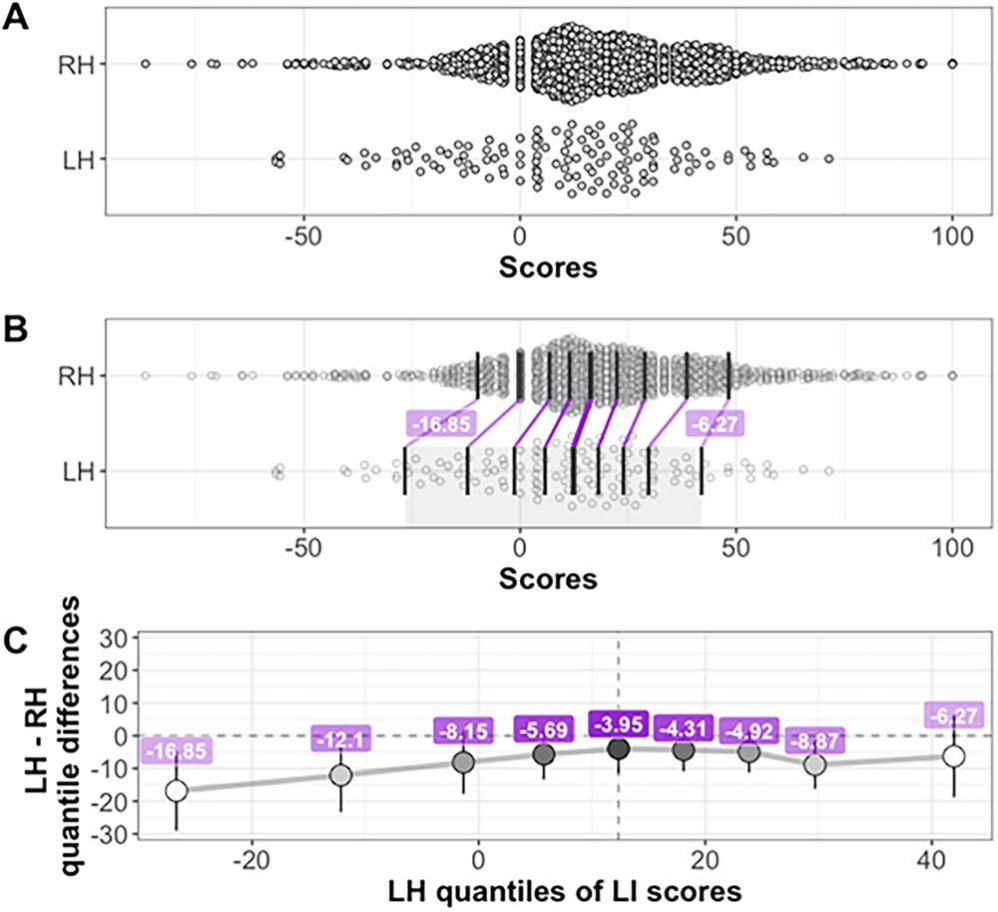
Scatterplots and shift function for Bergen DL LI scores. Panel a shows the distribution of LH and RH scores. Panel B illustrates the same distributions. The dark vertical lines mark the deciles in each distribution, with the median line of each distribution slightly thicker. Between distributions, the matching deciles are joined by purple lines, indicating a negative decile difference between the LH and RH groups. The values of the differences for deciles 1 and 9 are indicated in the superimposed labels. In panel C, the *x*-axis shows the deciles of LH scores, and the *y*-axis the differences between deciles (how much LI score deciles from the RH distribution needs to be shifted to match those of the LH distribution). The vertical lines indicate the 95% bootstrap confidence intervals. These negative LI quantile difference scores indicate that the left-handed group have smaller LIs at all points of the distribution.

The deciles of the two separate handedness LI distributions were compared using a shift function (*k* = 2,000), displayed in Fig. 2. To match the two distributions, right-handed scores needed to be consistently shifted negatively (towards the left-eared end). The largest difference between the distributions is in the left tail, with the first and second decile having confidence intervals, which suggests that the two distributions differ. A difference between the groups can also be seen at decile 8 (second from the right). When a confidence interval does not include zero, the difference is considered significant with an alpha threshold of 0.05 (Rousselet et al. 2017). The nonlinearity of the shift function suggests that there are asymmetric differences in the deciles of the two distributions.

### Bangor dichotic listening LI scores

A REA was found for both right-handers (*M* = +25.06, *SD* = 27.16, 95% CI +22.86, +27.28), *t*(587) = 22.38, *P* < 0.001, and left-handers (*M* = +19.78, *SD* = 30.11, 95% CI +17.00, 22.56), *t*(453) = 14.00, *P* < 0.001. Right-handed participants were, on average, found to have a higher LI score compared to left-handers, *t*(1,040) = 2.97, *P* = 0.003, *d* = 0.18, 95% CI of the mean difference [1.74, 8.82]. The proportions of REA were compared in the two groups. Two left-handers had no ear advantage and were excluded from this analysis. As above, 84.86% of the right-handers (499/588) and 80.31% of the left-handers (363/452) had right ear advantages, and this difference in proportions was statistically significant using an α of 0.05, *z* = 1.93, *P* = 0.027 (one-tailed), *h* = 0.12, 95% of the difference [−0.1%, 9.3%].

**Fig. 3:**
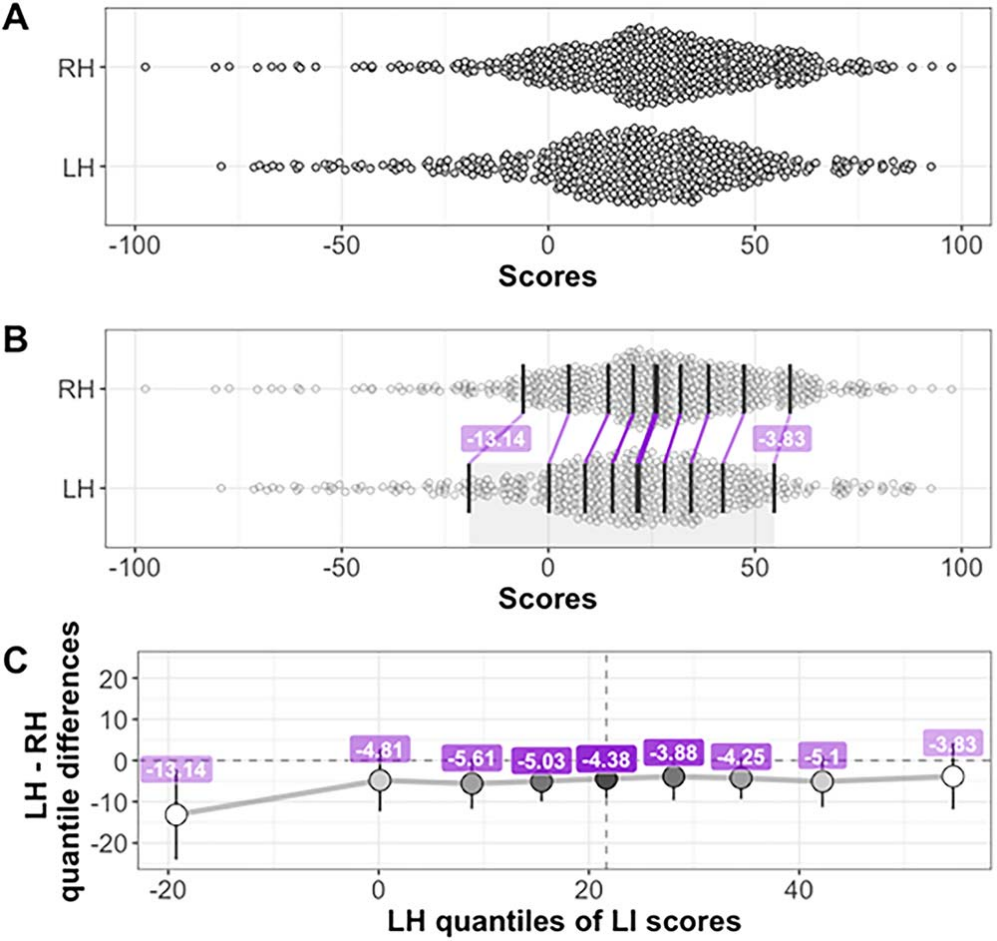
Scatterplot and shift function for Bangor DL LI scores. Panel a shows the distribution of LH and RH scores. Panel B illustrates the same distributions. The dark vertical lines mark the deciles in each distribution, with the median line of each distribution slightly thicker. Between distributions, the matching deciles are joined by purple lines, indicating a negative decile difference between the LH and RH groups. The values of the differences for deciles 1 and 9 are indicated in the superimposed labels. In panel C, the *x*-axis shows the deciles of LH, and the *y*-axis the differences between deciles (how much deciles from the RH distribution need to be shifted to match those of the LH distribution). The first and fourth deciles significantly differed in the two groups.

The shift function (*k* = 2,000) can be seen in Fig. 3 with all scores slightly negatively shifted. The largest differences between the distributions can be seen in the far-left tail, with the first (and fourth) decile having confidence intervals that suggests that the two distributions differ significantly.

## Discussion

The aim of the current study was to compare distributions of dichotic listening scores in two large samples of right-handers and left-handers. It was predicted that there would be differences in the shapes of the distributions that may not be detected by measures of central tendency alone. In both of these two large samples, there was a difference in average scores, with right-handers having a higher LI as compared to left-handers. When a shift function was used to compare the deciles in the two distributions, the differences in the Bergen sample scores at the first, second and eighth decile were statistically significant. In the Bangor sample, the first and fourth deciles were statistically significant.

These data support our hypothesis that subtle differences in the distributions of DL scores for right- and left-handers may be at least partially responsible for the variability of group differences in previous studies. Differences between the two distributions are not statistically significant at all points, but it may be telling that the numerically largest differences in both samples are from the first decile, the LEA end of the distribution. These are also statistically significant in the frequentist sense in both groups. This finding is particularly interesting as the Bangor group have some data which suggest that an LEA on CV dichotic listening, coupled with strong left sidedness in general, could be useful in predicting the rare, atypical right-hemispheric specialization for language (see Hunter and Brysbaert 2008, and Van der Haegen et al. 2011, for the suggestion that this type of prediction may also work with visual asymmetries). Recently, Sørensen and Westerhausen (2020) have modeled how to estimate hemispheric asymmetry for language based on dichotic listening scores using parts of the data from Bangor and Bergen, as well as data from Bless et al. 2015. They found that ear scores could provide sensible measures of the likelihood of right- or left-brain specialization in individual participants, in that they match the well-established estimates from the literature on language and handedness.

Our results paint a different picture to that of Packheiser et al. (2020), one of few studies with a larger sample size, comparing dichotic listening scores for the different handedness groups. The approach taken here is different to Packheiser et al., as they correlated dichotic listening scores with EHI handedness scores. When handedness was treated as a continuous variable in this way, they found little to no evidence for a relationship between EHI and dichotic listening scores. Independently, two referees suggested more direct comparisons of our data with that of Packheiser et al. (2020), for which Packheiser and colleagues kindly provided their raw data. Using their data, we performed the same shift function analysis as a function of handedness group. Although the quantile differences all have confidence intervals that overlap zero, the shift function suggests the same effects as seen in sample 1 and sample 2 of the present paper; the largest numerical difference in the first decile, suggesting increased left-hand representation in the LEA end of the distribution.

In addition, we calculated the correlation coefficient between handedness questionnaire scores and DL LI scores for the Bangor data (the only dataset with handedness questionnaire scores available for participants), as Packheiser et al. (2020) did with their sample. Unsurprisingly, there is a weak, but statistically significant, correlation, *r*(1,018) = 0.116, *P* < 0.001, 95% CI [.055, 0.176] (see supplementary materials 1) similar in magnitude to that reported in Pakheiser et al. (*r* = 0.063, *P* < 0.05, 95% CI [.013, 0.112]). We also report this correlation separately for right- and left-handed participants from both studies (using 0 as a cut-off for group membership) in the supplemental materials.

Of course, correlating handedness scores with dichotic listening scores is conservative. One problem is skew in handedness scores as measured by the EHI or our WHQ. In addition, in random samples such as those used in Packheiser et al. (2020), the number of left-handers will inevitably be rather low (137 in their data out of 1,554). Distribution data with these kinds of variables can be useful in interpreting correlation coefficients which are numerically small but statistically significant, as these can be driven, in part, by differing subgroups hidden within the sample that are overrepresented at different ends of one of the two variables being correlated.

The two samples in the current study (Ns = 1,358 and 1,042) are two of the largest lab-based dichotic listening studies to compare handedness differences, and to our knowledge this is the first ever study to compare the full distribution of scores between the two groups. Additionally, one of the samples is enriched with left-handers (about 40%). Of course, one limitation may be that the tails of the distribution consist of more individuals with large hearing differences between their ears, as most dichotic listening studies do not measure these ear differences. Hugdahl et al. (2008) found that interaural intensity differences of less than about 10 dB are unlikely to significantly shift ear advantages in a sample of right-handed listeners. Many large-scale studies of sensory hearing loss do not provide information about frequency of deficits in the right or left ears but we have been informed that, unsurprisingly, they tend to be distributed evenly between left and right (Y. Agrawal, personal communication, 17 June, 2020). In other words, hearing differences between ears in individual participants are at worst a source of randomly distributed noise: they are no more likely to affect either end of any dichotic listening LI distribution. In addition, many of the participants in the Bergen database underwent audiometry for interaural sound threshold differences. When the difference between the two ears was large (>10 dB), participants were discarded.

We have already commented on reasonably good reliabilities of test–retest of verbal dichotic listening including the CV task used here. Recent analyses by Westerhausen and his collaboratories suggest three blocks of 30 trials or more would be best practice for use of DL as one tool in investigating underlying cerebral asymmetry for language-related processes. Sørensen and Westerhausen (2020) have used dichotic listening data from people with known hemispheric specialization, assessed by word generation, to produce a Bayesian model that predicts language laterality. Their current model suggests using an LI cutoff of greater than −0.10 suggests an 80% chance of being left dominant for language. Using more trials also reduces the proportion of cases that cannot be classified as left or non-left dominant. Another paper from the same team (Westerhausen and Samuelsen 2020) has evaluated an optimized version of CV dichotic listening where only voiced or unvoiced consonant pairs are presented to the participants. Test–retest reliabilities (estimated from intra-class correlation coefficients) go from 0.65 for one 40-trial block, to 0.86 for two blocks, to 0.88 for three blocks. Taken together, these results suggest some merit to the Bangor approach of using three blocks of unforced trials for experiments focused on language laterality.

The findings from our study are relevant for both practical and theoretical reasons. Surprisingly very few contemporary models in neurobiology of language make predictions regarding hemispheric specialization of different language processes (for a summary see Bradshaw et al. 2017a). Of the few that do, right-ear advantages on verbal dichotic listening tasks are hard to reconcile with claims that early processes in speech perception are bilateral and not lateralized to the left hemisphere. For example, Hickok and Poeppel’s (2007) well-regarded model suggests that early speech processing, such as syllable perception, would be bilaterally represented in so-called parabelt auditory cortices. In their account only later processes, closer to speech output are left-lateralized in right-handers. In this account why most people have an REA is mysterious. However, it may be that Kimura’s (1967) structural model is relevant here: only under dichotic conditions such as those used with the CV dichotic listening test are ipsilateral acoustic pathways suppressed. In most imaging studies and of course in everyday speech, dichotic conditions are not the norm and early speech processing may indeed be largely bilateral. And of course, in dichotic listening tasks central nervous systems are confronted with the necessity of making a choice between competing inputs; it may be that top–down mechanisms involving the frontal cortex play a role in the REA in left dominant participants. Practically speaking, our data suggest that dichotic listening could play a role in predicting language lateralization (for a similar argument about visual half field studies, see Gerrits et al. 2020). For example, it could prove useful as part of a pre-screening process used to identify people likely to show typical or atypical hemispheric specialization. Much of the work on this question has come from epilepsy research, where fMRI, as an alternative to invasive sodium amytal testing, has been the norm for a number of years now. Recently, a few groups, including ours, have shown renewed interest in the relatively neglected right-dominant participants (Badzakova-Trajkov et al. 2010; Tzourio-Mazoyer et al. 2016; Gerrits et al. 2019; Karlsson et al. 2022) outside of this largely clinical context.

A caveat to be added here is that in the neuropsychology literature, speech, and language lateralization are referred to interchangeably. This tendency follows from the rather consistent suggestions of left hemisphere specialization for speech production (Broca’s and Transcortical motor aphasias), speech perception/comprehension (Wernicke’s and Transcortical sensory aphasias; dichotic listening), reading (alexia; fMRI and the visual Word Form Area), and of course writing (agraphia). Nevertheless, cognitive neuroscientists have documented rather distinct neural circuits for different components of language, for example speech perception and speech comprehension (contrast Norman-Haignere et al. 2015; Overath et al. 2015; Guenther 2016; with Fedorenko et al. 2010, 2011). These different circuits could, in theory, have different lateralization profiles, but this intriguing question tends not to be the focus of many such experiments. In fact, the dependence on group averages which require choice of a particular statistical threshold is not optimal for characterizing lateralization breadth or depth (Bradshaw et al. 2017b; Johnstone et al. 2020). Another caveat is that these studies sample only right-handers (Willems et al. 2014; Bailey et al. 2020). These circuits, and speech production itself (surprisingly bilateral in threshold-dependent group averages) would benefit from study using a threshold-independent lateralization measure in individual participants, and to include participants likely to have less “typical” hemispheric specialization for at least one language or speech-related function. A few groups (including ours) have done so by oversampling left-handers (Badzakova-Trajkov et al. 2010; Van der Haegen et al. 2011; Mazoyer et al. 2016; Woodhead et al. 2021; Johnstone et al. 2020, 2021; Karlsson et al. 2022; Petit et al. 2023), as we have done here in the Bangor sample with dichotic listening.

Dichotic listening has proved a useful tool outside of the hemispheric specialization studies that it so quickly came to dominate in the 1980s. Despite this ongoing work on top-down executive processes, we think that dichotic listening as a tool for examining asymmetry has not worn out its usefulness yet (see also Sørensen and Westerhausen 2020). Both samples in the current study demonstrate that there are subtle differences in the distribution of dichotic listening scores that nicely mirror differences in hemispheric specialization for language, and are likely behind the small average difference not always found between right- versus left-handers. The data here, coupled with those of Sørensen and Westerhausen (2020) and arguments for optimization of DL by Westerhausen and Samuelsen (2020), suggest that it might be time to have another look at the affordance that this simple, easy to use paradigm provides.

## Supplementary Material

Karlsson_etal_DLdistribusions_Supplemental_material_tgad009

Datasets_link_tgad009

## Data Availability

The data underlying this article will be shared on reasonable request to the corresponding author.
