# Supplementary material for: Analysis of distributions reveals real differences on dichotic listening scores between left- and right-handers
Source: Cereb Cortex Commun. 2023 Jun 1;4(2):tgad009. doi: 10.1093/texcom/tgad009 (PMC10262840; doi:10.1093/texcom/tgad009)
Supplement: Karlsson_etal_DLdistribusions_Supplemental_material_tgad009 [file karlsson_etal_dldistribusions_supplemental_material_tgad009.docx]

Supplemental materials

**Analysis of distributions reveals real differences on dichotic listening scores between left- and right-handers**

Emma M. Karlsson^a,b^, Kenneth Hugdahl^c^, Marco Hirnstein^c^ and David P. Carey ^a^

^a^Institute of Cognitive Neuroscience, School of Human and Behavioural Sciences, Bangor University, Bangor, UK

^b^ Department of Experimental Psychology, Ghent University, Ghent, Belgium

^c^Department of Biological and Medical Psychology, University of Bergen, Bergen, Norway

**Author Note**

Emma M. Karlsson
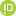
 https://orcid.org/0000-0002-9605-1923

Kenneth Hugdahl 
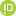
 https://orcid.org/0000-0002-0008-4326

Marco Hirnstein 
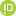
 https://orcid.org/0000-0002-6291-0929

David P. Carey
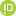
 https://orcid.org/0000-0001-8860-9731

We have no known conflict of interest to disclose.

Correspondence concerning this article should be addressed to David P. Carey, School of Human and Behavioural Sciences, Bangor University, Bangor, UK, LL57 2AS. Email: d.carey@bangor.ac.uk

**Figure 1.** Scatterplot depicting the relationship between dichotic listening (DL) laterality index (LI) score and Waterloo handedness questionnaire (WHQ) score for the Bangor sample.


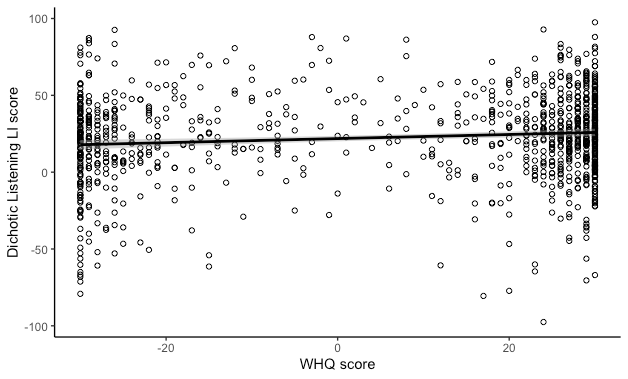


**Figure 2.** Scatterplot depicting the relationship between DL LI score and WHQ for the right-handers in the Bangor sample (using a WHQ score of >0 as cut-off).

**
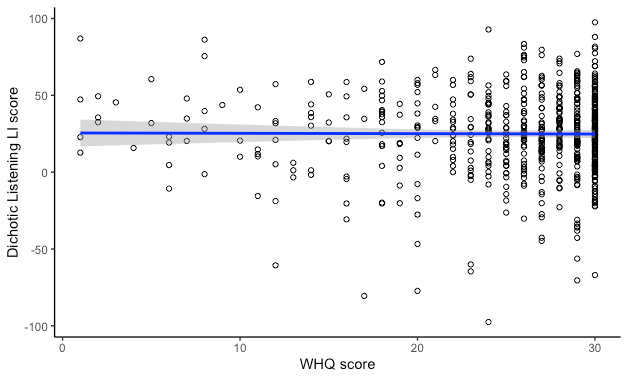
**

**Figure 3.** Scatterplot depicting the relationship between DL LI score and WHQ for the left-handers in the Bangor sample (using a WHQ score of < or = 0 as cut-off).

**
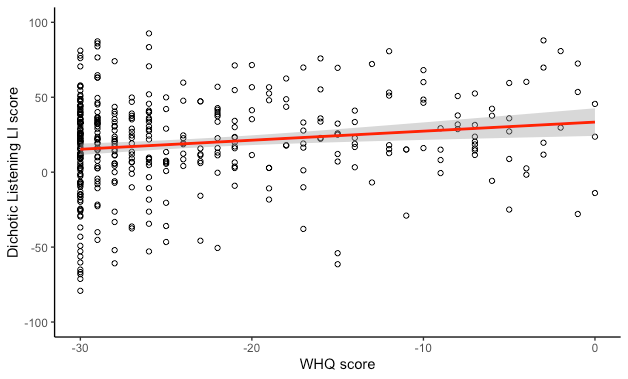
**

**Figure 4.** Scatterplot depicting the relationship between dichotic listening (DL) laterality index (LI) score and Edinburgh handedness inventory (EHI) score for the Packheiser et al. (2020) sample.

**
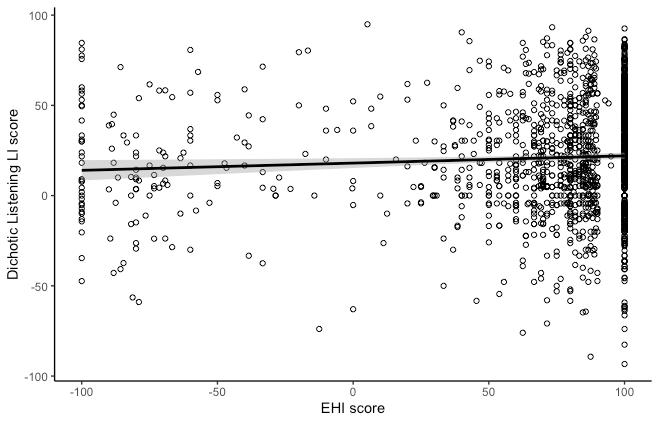
**

**Figure 5.** Scatterplot depicting the relationship between DL LI score and EHI for the right-handers in the Packheiser et al. (2020) sample (defined as EHI > 0.1).

**
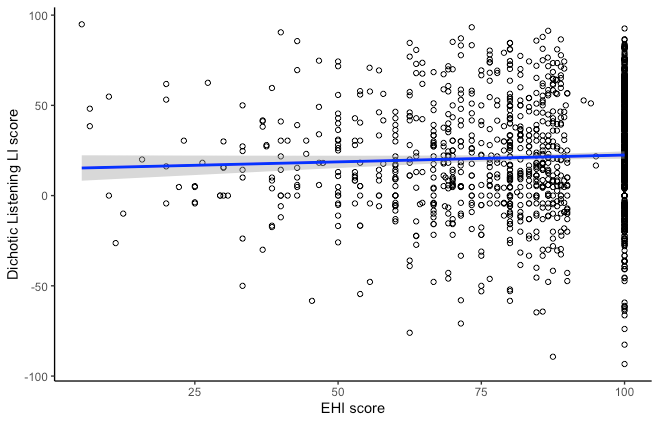
**

**Figure 6.** Scatterplot depicting the relationship between DL LI score and EHI for the left-handers in the Packheiser et al. (2020) sample (defined as EHI < or = 0).

**
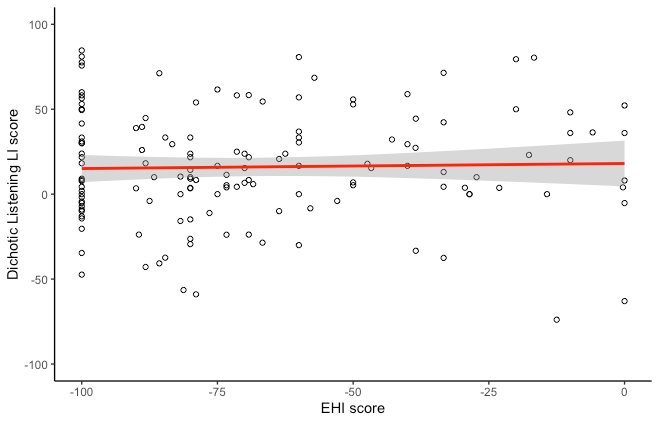
**

**Figure 7.** Scatterplots and shift function for Packheiser ei al. (2020) DL LI scores

**
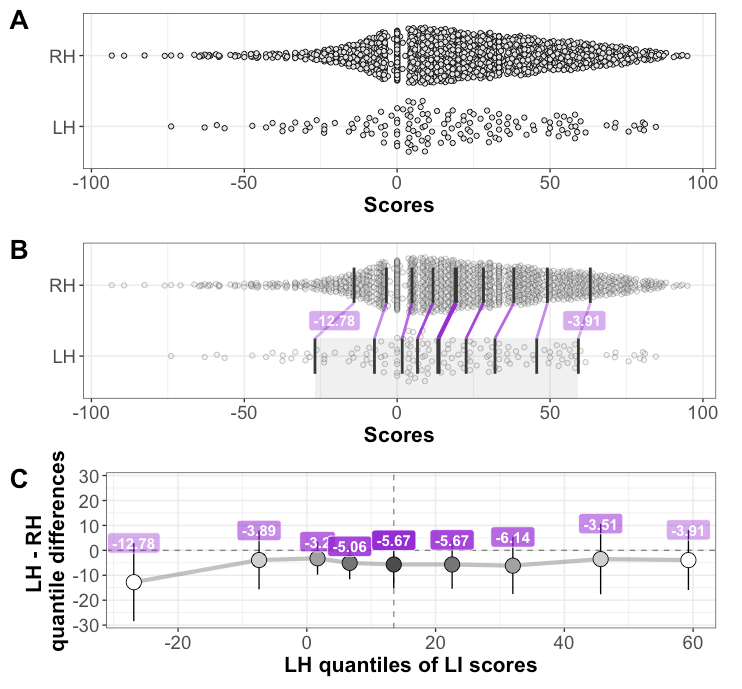
**

*Note.* Panel A shows the distribution of left-handed (LH) and right-handed (RH) scores. Panel B illustrates the same distributions. The dark vertical lines mark the deciles in each distribution, with the median line of each distribution slightly thicker. Between distributions, the matching deciles are joined by purple lines, indicating a negative decile difference between the LH and RH groups. The values of the differences for deciles 1 and 9 are indicated in the superimposed labels. In Panel C, the x-axis shows the deciles of LH scores, and the y-axis the differences between deciles (how much LI score deciles from the RH distribution needs to be shifted to match those of the LH distribution). The vertical lines indicate the 95% bootstrap confidence intervals. These negative LI quantile difference scores indicate that the left-handed group have smaller LIs at all points of the distribution.

*Table 1.* Shift function statistics for each of the bootstrapped deciles in the Bergen DL data for right-handers (RH) and left-handers (LH) with the difference in scores at each decile, and 95% confidence intervals of the difference.

| Decile | LH LI | RH LI | Difference | Lower CI | Upper CI |
| --- | --- | --- | --- | --- | --- |
| 0.1 | -26.69 | -9.84 | -16.85 | -29.02 | -4.68 |
| 0.2 | -12.17 | -0.08 | -12.10 | -23.33 | -0.87 |
| 0.3 | -1.35 | 6.80 | -8.15 | -17.81 | 1.50 |
| 0.4 | 5.74 | 11.43 | -5.69 | -13.41 | 2.02 |
| 0.5 | 12.34 | 16.29 | -3.95 | -11.46 | 3.56 |
| 0.6 | 18.08 | 22.39 | -4.31 | -10.92 | 2.31 |
| 0.7 | 23.87 | 28.78 | -4.92 | -11.30 | 1.47 |
| 0.8 | 29.68 | 38.55 | -8.87 | -16.24 | -1.50 |
| 0.9 | 41.95 | 48.22 | -6.27 | -18.80 | 6.26 |

*Table 2.* Shift function statistics for each of the bootstrapped deciles in the Bangor DL data for right-handers (RH) and left-handers (LH) with the difference in scores at each decile, and 95% confidence intervals of the difference.

| Decile | LH LI | RH LI | Difference | Lower CI | Upper CI |
| --- | --- | --- | --- | --- | --- |
| 0.1 | -19.26 | -6.12 | -13.14 | -24.09 | -2.18 |
| 0.2 | 0.12 | 4.93 | -4.81 | -12.37 | 2.74 |
| 0.3 | 8.86 | 14.47 | -5.61 | -11.74 | 0.52 |
| 0.4 | 15.49 | 20.52 | -5.03 | -9.94 | -0.11 |
| 0.5 | 21.66 | 26.04 | -4.38 | -9.11 | 0.34 |
| 0.6 | 28.06 | 31.94 | -3.88 | -9.65 | 1.88 |
| 0.7 | 34.47 | 38.72 | -4.25 | -9.36 | 0.86 |
| 0.8 | 42.20 | 47.29 | -5.10 | -11.35 | 1.16 |
| 0.9 | 54.62 | 58.46 | -3.83 | -11.88 | 4.21 |

*Table 3.* Shift function statistics for each of the bootstrapped deciles in the Packheiser et al. (2020) data for RH and LH (using an EHI of 0 as the cut-off), with the difference in scores at each decile, and 95% confidence intervals of the difference.

| Decile | LH LI | RH LI | Difference | Lower CI | Upper CI |
| --- | --- | --- | --- | --- | --- |
| 0.1 | -26.88 | -14.10 | -12.78 | -27.88 | 2.32 |
| 0.2 | -7.42 | -3.53 | -3.90 | -15.71 | 7.92 |
| 0.3 | 1.66 | 4.86 | -3.20 | -9.59 | 3.19 |
| 0.4 | 6.66 | 11.71 | -5.06 | -11.53 | 1.42 |
| 0.5 | 13.50 | 19.17 | -5.67 | -15.31 | 3.98 |
| 0.6 | 22.56 | 28.23 | -5.67 | -15.52 | 4.18 |
| 0.7 | 32.00 | 38.14 | -6.14 | -17.21 | 4.92 |
| 0.8 | 45.64 | 49.15 | -3.51 | -17.49 | 10.47 |
| 0.9 | 59.27 | 63.17 | -3.91 | -16.05 | 8.24 |
