# Supplementary material for: Analysis of distributions reveals real differences on dichotic listening scores between left- and right-handers
Source: Cereb Cortex Commun. 2023 Jun 1;4(2):tgad009. doi: 10.1093/texcom/tgad009 (PMC10262840; doi:10.1093/texcom/tgad009)
Supplement: Datasets_link_tgad009 [file datasets_link_tgad009.docx]

Datasets for the paper can be found using the following link: [https://osf.io/zc78u/?view_only=ae9a934e4a9c426b8d184239fbd19429](https://nam12.safelinks.protection.outlook.com/?url=https%3A%2F%2Fosf.io%2Fzc78u%2F%3Fview_only%3Dae9a934e4a9c426b8d184239fbd19429&data=05%7C01%7Cmichelle.pizzuti%40yale.edu%7C3f1859b5889344b7ed7608dac0c66f27%7Cdd8cbebb21394df8b4114e3e87abeb5c%7C0%7C0%7C638034255766089077%7CUnknown%7CTWFpbGZsb3d8eyJWIjoiMC4wLjAwMDAiLCJQIjoiV2luMzIiLCJBTiI6Ik1haWwiLCJXVCI6Mn0%3D%7C3000%7C%7C%7C&sdata=FKe8pO8R9yqbETwsoxCZdbnrPn5fSoSn%2BIVbV636LG4%3D&reserved=0)
